# Characterization of Chemical Suicides in the United States and Its Adverse Impact on Responders and Bystanders

**DOI:** 10.5811/westjem.2016.9.32267

**Published:** 2016-10-07

**Authors:** Ayana R. Anderson

**Affiliations:** Agency for Toxic Substances and Disease Registry, Atlanta, Georgia

## Abstract

**Introduction:**

A suicide trend that involves mixing household chemicals to produce hydrogen sulfide or hydrogen cyanide, commonly referred to as a detergent, hydrogen sulfide, or chemical suicide is a continuing problem in the United States (U.S.). Because there is not one database responsible for tracking chemical suicides, the actual number of incidents in the U.S. is unknown. To prevent morbidity and mortality associated with chemical suicides, it is important to characterize the incidents that have occurred in the U.S.

**Methods:**

The author analyzed data from 2011–2013 from state health departments participating in the Agency for Toxic Substances and Disease Registry’s National Toxic Substance Incidents Program (NTSIP). NTSIP is a web-based chemical incident surveillance system that tracks the public health consequences (e.g., morbidity, mortality) from acute chemical releases. Reporting sources for NTSIP incidents typically include first responders, hospitals, state environmental agencies, and media outlets. To find chemical suicide incidents in NTSIP’s database, the author queried open text fields in the comment, synopsis, and contributing factors variables for potential incidents.

**Results:**

Five of the nine states participating in NTSIP reported a total of 22 chemical suicide incidents or attempted suicides during 2011–2013. These states reported a total of 43 victims: 15 suicide victims who died, seven people who attempted suicide but survived, eight responders, and four employees working at a coroner’s office; the remainder were members of the general public. None of the injured responders reported receiving HazMat technician-level training, and none had documented appropriate personal protective equipment.

**Conclusion:**

Chemical suicides produce lethal gases that can pose a threat to responders and bystanders. Describing the characteristics of these incidents can help raise awareness among responders and the public about the dangers of chemical suicides. Along with increased awareness, education is also needed on how to protect themselves.

## INTRODUCTION

In 2007, Japan documented the first reports of chemical or detergent suicides, and 2,000 such suicides have been reported there since then.^1^ Around the same time, incidents of chemical suicide, also known as detergent or hydrogen sulfide suicide, were reported in the United States (U.S.).^2^–^4^ Internet websites provide detailed instructions on how to commit suicide by mixing household chemicals usually to produce hydrogen sulfide or hydrogen cyanide gas in an enclosed space.^1^,^2^ Hydrogen sulfide is a colorless gas that is heavier than air, has a sweetish taste, and smells like rotten eggs.^5^ Hydrogen cyanide gas has a faint, bitter almond odor and bitter burning taste.^3^ High-level exposure to either chemical could result in immediate death.^3^,^5^

Because no one database is responsible for tracking chemical suicides in the U.S., the actual number of incidents is unknown. In 2011, using National Vital Statistics System (NVSS) and Google searches, Reedy, Schwartz, and Morgan found that 30 chemical suicides occurred in the U.S. from 2008–2010.^4^ Medical examiners confirmed the chemical suicides found in the NVSS.^4^ In 2011, using chemical surveillance systems the Agency for Toxic Substance and Disease Registry (ATSDR) reported 10 chemical suicide incidents; however, this report focused only on incidents that occurred in vehicles.^6^ The report also showed that chemical suicides were a threat not only to the person committing suicide, but to responders and innocent bystanders as well. Both reports indicated that their findings were most likely underestimates of the true frequency of chemical suicide incidents.^4^,^6^

Our report updates ATSDR’s chemical suicide data by including three additional years of surveillance data (2011–2013) and other locations where chemical suicide incidents occurred. Through describing the characteristics of these incidents, we hope to raise awareness about potential exposure risks to responders and bystanders from chemical suicides incidents so that recommendations for preventative actions can be made to avoid exposure and exposure-related injuries associated with chemical suicides.

## METHODS

This report used data from nine state health departments participating in ATSDR’s chemical incident surveillance system, the National Toxic Substance Incidents Program (NTSIP), to determine the frequency of chemical suicides occurring during 2011–2013 (Figure 1). NTSIP is a web-based system that tracks the public health consequences (e.g., morbidity, mortality) from acute chemical releases. Reporting sources for NTSIP incidents typically include first responders, hospitals, state environmental agencies, and media outlets. The type of data NTSIP collects includes but is not limited to time, date, and day of the week event occurs, geographical location, factors contributing to the release, specific information on injured persons (age and sex), and type of personal protective equipment (PPE). For more information about the NTSIP database go to https://www.atsdr.cdc.gov/ntsip/state_partners.html. To identify chemical suicide incidents, open text fields were queried in the NTSIP comment, synopsis, and contributing factors variables for the following terms: *suicide, intentional, inhaled, death, die, kill, detergent*, and *household chemical*. Using SAS, we performed descriptive analyses to describe the chemical suicide incidents and identify the public health impact.

## RESULTS

During 2011–2013, participating states reported a total of 9,398 acute chemical releases in the NTSIP database. Five states reported 22 chemical suicides or attempted suicides during 2011–2013 (Figure). Most of these incidents (95.5%, n=21) occurred in enclosed areas (i.e., vehicles, hotel rooms, bathrooms, or other rooms in a house). One incident occurred in a more open space where chemicals were mixed in a parking lot of a hardware store. These 22 chemical suicide incidents affected a total of 43 victims: 15 suicide victims who died, seven people who attempted suicide but survived, eight responders, and four employees at the coroner’s office; the remaining nine were members of the general public or unknown victims (Table). The most frequently reported injuries were respiratory irritation, shortness of breath, and headaches. Of the 22 incidents, nine (41%) reported decontamination of victims, either on scene, at the medical facility, or at both locations. Of those nine incidents, one reported decontamination of additional victims but not the suicide victim (Table ). None of the injured responders reported being a certified HazMat technician (one who is trained to handle hazardous materials (e.g. chemicals) or wearing appropriate PPE. Seven of the incidents included evacuations (Table). Approximately 54.5% of the incidents resulted in hydrogen sulfide releases, 18.1% resulted in chlorine gas, and none resulted in the release of hydrogen cyanide.

### Illustrative Case Reports

#### New York

In 2013, the police department, the fire department, a HazMat team, local emergency management services, and the coroner’s office responded to a chemical suicide that occurred in a private residence where the victim had mixed chemicals to produce hydrogen sulfide gas. Two police officers were exposed at the scene of the incident. The victim’s body was transported to the coroner’s office where off-gassing occurred and four employees were exposed. The following injuries were reported for exposed coroner employees (who were not at the scene): central nervous system issues, headaches, shortness of breath, and gastrointestinal problems. Responders suffered from central nervous issues, respiratory issues, skin irritation and headaches. Responders did not wear PPE or conduct decontamination procedures.

#### Tennessee

In 2011, an individual parked outside an outlet store, combined chemicals in a closed car, creating hydrogen sulfide gas. A warning sticker the victim had left on the vehicle alerted responders to a chemical hazard. The local fire department decontaminated the body and scene. No additional injuries were reported involving first responders.

## DISCUSSION

Even though chemical suicides accounted for <1% of all incidents reported to NTSIP, they may have serious outcomes. Secondary contamination is a major concern associated with chemical suicides. This report of 22 incidents found an additional 21 people that were injured in addition to the person who committed or attempted to commit suicide by means of chemical release. As the New York case illustrates, if a person’s skin or clothes are exposed to hydrogen sulfide, then others who come into contact with that person (bystanders or responders) can experience secondary contamination through off-gassing.^3^,^5^ HazMat training and wearing proper PPE can help prevent secondary exposure in responders.^7^ Data in this report showed that none of the first responders were HazMat technician certified nor did they wear PPE when responding to the incidents. Therefore, training first responders to recognize a chemical suicide attempt and to take proper exposure precautions could reduce injuries associated with such incidents.

Another measure responders could take to prevent secondary exposure due to off-gassing is proper decontamination of the scene, exposed individuals, and corpses.^3^,^6^,^8^ Rapidly removing contaminated clothing, flushing skin and hair with plain water for two to three minutes, and then washing with mild soap^3^ can prevent further injuries for bystanders and responders who have been exposed. Double-bagging contaminated clothing, personal belongings, and corpses 3,6 can also prevent secondary exposure, as can transporting exposed individuals in a well-ventilated vehicle.^6^

Responders must be able to recognize signs that a chemical suicide has taken place so that they do not enter the hazardous environment unprepared. In some incidents, victims placed signs to warn that hazardous substances were on the premises.^6^ Responders should survey the surroundings for any other visible signs that suggest a chemical suicide, such as open containers or attempts to seal windows, doors, or vents with tape.^8^ Responders who are not certified HazMat technicians should wait to enter the hazardous environment until a certified responder arrives.

## LIMITATIONS

The findings in this report are subject to two limitations. First, due to the limited number of states participating in NTSIP, the data might not be generalizable to the entire U.S. Second, the number of chemical suicides reported in this analysis is most likely an underestimate; some suicides may not have been reported or may have been missed through the key word searches.

## CONCLUSION

Chemical suicide environments can pose a threat to responders and bystanders. Education is essential to raise awareness among responders and the public about the dangers of chemical suicides. Responders should be trained to recognize chemical suicide scenarios and to follow protocols to decrease exposure risk, including the appropriate use of PPE and decontamination of exposed persons including corpses. Members of the public also need to be able to identify chemical suicide situations and take steps to ensure their personal safety. Additionally, ongoing education and outreach efforts targeted to healthcare providers, public health practitioners, and others may lead to better strategies to recognize and prevent chemical suicide incidents.

## Figures and Tables

**Figure:**
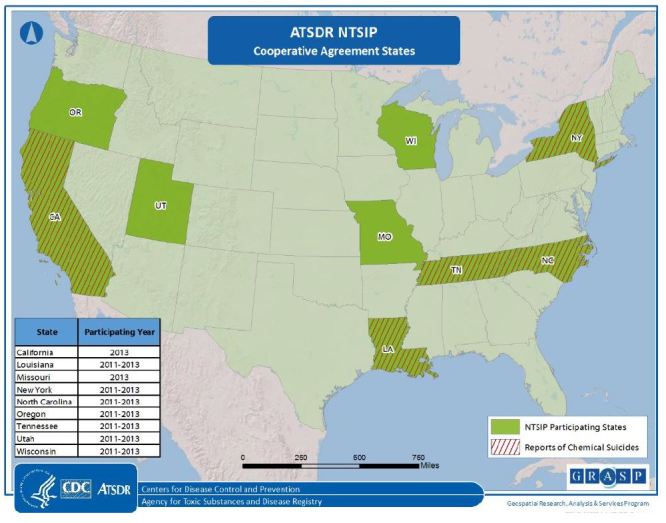
Participating National Toxic Substance Incidents Program (NTSIP) states and states reporting chemical suicides, NTSIP 2011–2013.

**Table:** Chemical suicides case characteristics, National Toxic Substance Incidents Program (NTSIP) 2011–2013.

Year	Incident location	Evacuation ordered?	Additional victims?	Victim decontamination location
2011	Vehicle	No	No	Scene
2011	Vehicle	No	No	Scene
2011	Vehicle	No	No	Scene/Medical facility
2011	Vehicle	Yes	Yes;1 emergency medical technician;1 police officer	Unknown*
2011	Vehicle	No	Yes; 2 police officers; 1 member of the general public	Scene (suicide victim was not decontaminated)
2011	Vehicle	No	Yes; 2 members of the general public	Scene/Medical facility
2011	Vehicle	No	No	Medical facility
2011	Vehicle	No	No	Medical facility
2012	Home	No	No	None
2012	Vehicle	No	No	None
2012	Unknown	No	No	Scene
2012	Vehicle	No	Yes; 1 member of the general public	None
2012	Vehicle	No	No	None
2012	Hotel	Yes	No	None
2013	Vehicle	No	Yes; 1 police officer	Unknown*
2013	Vehicle	Yes	No	Scene
2013	Hotel	Yes	No	None
2013	Dormitory room	Yes	Yes; 1 employee emergency responder; 5 unknown	None
2013	Home	Yes	Yes; 4 employees at coroner’s office; 2 police officers	None
2013	Hardware store	No	No	Unknown*
2013	Campground	Yes	No	None
2013	Vehicle	No	No	None
